# Draft Genome Sequence of the Tomato Stem Endophyte Bacillus safensis TS3

**DOI:** 10.1128/mra.00816-22

**Published:** 2022-10-27

**Authors:** Vladimir K. Chebotar, Maria S. Gancheva, Gerben P. Voshol, Natalia V. Malfanova, Evgenyi S. Karasev, Elena P. Chizhevskaya, Alexander N. Zaplatkin, Alexander V. Khiutti, Alexander M. Lazarev, Nadim M. Gadjiev, Vera A. Lebedeva, Svetlana V. Balakina

**Affiliations:** a All-Russia Research Institute of Agricultural Microbiology, St. Petersburg, Russia; b Department of Genetics and Biotechnology, Faculty of Biology, Saint Petersburg State University, Saint Petersburg, Russia; c Institute of Biology Leiden, Leiden, Netherlands; d All-Russian Research Institute of Plant Protection, St. Petersburg, Russia; e Leningrad Research Institute for Applied Agricultural Science “Belogorka,” Leningrad Region, Russia; Indiana University, Bloomington

## Abstract

Tomato stem endophyte Bacillus safensis TS3 was isolated from surface-sterilized stems of greenhouse tomato plants. Here, we sequenced the complete genome of this strain to understand the molecular mechanisms underlying its beneficial activities.

## ANNOUNCEMENT

Tomato stem endophyte Bacillus safensis TS3 was isolated from surface-sterilized stems of greenhouse tomato (Lycopersicon esculentum Mill.) plants cv. Carmello grown in salty soils. Bacterial isolation was performed as described previously (1). Strain TS3 was grown in LB medium at 30°C overnight. The total cellular DNA was isolated using the cetyltrimethylammonium bromide (CTAB)-NaCl method (2). The pair-end library for Illumina sequencing was prepared using the Nextera DNA Flex library prep kit (Illumina, USA). The genome sequencing was performed using the Illumina MiSeq sequencing system by using the MiSeq reagent kit v3 (Illumina). A total of 578,778 reads were generated with an average length of 300 bp. An assessment of the sequence quality was accomplished using FastQC (version 0.11.5) (3). Default parameters were used for all software unless otherwise specified. The Andrew And Aaron’s Awesome Assembly (A5) pipeline was used for sequence data cleaning, error correction, and assembly of TS3 (4). Genome annotation was carried out using the Prokka software package (5) and NCBI Prokaryotic Genome Annotation Pipeline (version 5.3) (6). Coding sequences were identified with Prodigal version 2.6.3 (7), and their putative function was elucidated using Blast+ searches against all the verified (on protein or transcript level) bacterial proteins from Uniprot. The remaining coding sequences with unknown function were subsequently scanned using hmmscan (version 3.2) against a series of HMM profile databases (Pfam version 29.0 [8] and TIGRFAMs version 15.0 [9], and transcription factor proteins were annotated at collecTF DB [10] and DBTBS version 5 [11]). rRNA and tRNA genes were located through RNAmmer (version 1.2) (12) and ARAGORN (13), respectively. The final assembly contains 3,716,624 bp in 34 contigs with an average G+C content of 41.7%. The maximum contig size is 821,011 bp and the *N*_50_ (median contig size) value was 354,550 bp. A total of 3,755 protein-coding sequences (CDS), 26 rRNAs, 81 tRNA genes, and 5 noncoding RNAs (ncRNAs) were predicted.

Identification of TS3 was done by using two alternative methods. First, we performed an average nucleotide identity (ANI) sequence analysis as described by reference 14. The resulting distance matrix was converted to a phylogenetic tree using the UPGMA method and visualized using FigTree (version 1.4.2) (15). Second, we performed a BLASTN search with the *gyrA* gene sequence and retrieved the top scoring hits. These sequences were subsequent aligned using Kalign (version 2) (16), and a maximum likelihood tree was constructed using FastTree (version 2) (17). The trees convincingly show that TS3 is grouped within the Bacillus safensis cluster. Six Bacillus safensis reference genomes (GenBank accession numbers NZ_CP010405, NZ_CP015607, NZ_CP015610, NZ_CP015611, NZ_CP018100, and NZ_CP018197) were used for comparative genome analyses. Comparative analysis of the genomes was performed using the cct program (version 1.0.3) (18). A genome comparison of the CDS of TS3 versus six closely related reference *B. safensis* genomes resulted in the identification of 115 proteins not present in the other six examined genomes. A large part of the genome of TS3 (>6%) is presumably dedicated to the production of various plant-growth-promoting and stress-alleviating metabolites. These metabolites include indoleacetic acid (IAA), volatiles, organic acids, vitamins, compatible solutes, exopolysaccharide (EPS), antioxidant enzymes, and metal reductases. In addition, TS3 has 10 antibiotic clusters (accounting for ~3% of the genome) presumably involved in the synthesis of antimicrobial peptides, including the biosurfactant pumilacidin and two different siderophores.

### Data availability.

The complete genome sequences were deposited in National Center for Biotechnology Information (NCBI) under the accession number JAIZBS000000000. The raw sequences was deposited in the Sequence Read Archive under the accession number SRR20622344 (BioProject PRJNA766560).
